# Leveraging AI to reduce operational healthcare costs: lessons from other industries

**DOI:** 10.1038/s44401-026-00070-7

**Published:** 2026-01-28

**Authors:** Andrew Wong, Brahmajee K. Nallamothu, Christopher A. Longhurst, Karandeep Singh

**Affiliations:** 1 University of Michigan Medical School, Ann Arbor, MI USA; 2 Seattle Children’s Hospital, Seattle, WA USA; 3 Division of Biomedical Informatics, Department of Medicine, University of California, San Diego, CA USA

**Keywords:** Business and industry, Computational biology and bioinformatics, Engineering, Health care, Mathematics and computing, Medical research

## Abstract

Despite the potential of AI to improve healthcare delivery, healthcare has lagged behind other sectors in its adoption of operational AI technologies. To address this gap, we outline examples of successful AI applications from other sectors, draw parallels to healthcare, and provide a roadmap for health system leaders to implement similar technologies. Effective integration will require tying AI initiatives to value and redesigning our health systems for AI-enabled workflows.

## Introduction

U.S. healthcare spending has risen to unprecedented levels, totaling $4.9 trillion in 2023 alone^1^. Novel technologies such as artificial intelligence (AI) have the potential to optimize clinical operations and reduce healthcare costs. The adoption of AI in healthcare, however, has lagged behind other industries^2,3^. This occurs despite the fact that the National Bureau of Economic Research has estimated that wider adoption of AI in the U.S. healthcare system could lead to savings between $200 billion and $360 billion annually^4^.

Until recently, health AI tools in the U.S. have primarily focused on clinical decision support, an area with active federal research funding and regulatory oversight from the Food and Drug Administration (FDA)^5,6^. While better diagnostic accuracy can reduce healthcare costs, this represents a relatively narrow slice of healthcare spending. Instead, operational and administrative activities such as workforce staffing, care coordination, billing and claims processing, and customer service contribute to a substantial portion of U.S. healthcare costs^7^, estimated to have been as high as $950 billion in 2019^8^. Many sectors outside of healthcare have found success in incorporating AI systems into their everyday operational workflows^9^, and AI tools have demonstrated returns on investment in areas such as retail, customer service, marketing, and aviation^10,11^. Within healthcare, early research has suggested a role for AI tools to shorten length of stay, reduce emergency department utilization, predict operational events such as hospital capacity surges, and automate administrative tasks such as billing, scheduling, and call center functions^12^.

Early successes have spurred a growing ecosystem of clinical AI tools developed by both well-established vendors and startup companies, but the rapid introduction of these tools has outpaced the evidence for their safety and efficacy. Reporting on operational AI outcomes is rare, leading to a paucity of academic literature, a lack of organized trials, and uneven adoption^13^. This raises concerns about the effectiveness of such AI tools and their role in healthcare^14^. Even when tools are effective, their use may lead to the unanticipated effect of driving up hidden healthcare costs. For instance, several large insurance companies have faced class action lawsuits for their use of AI to deny claims, an increasingly common practice that places additional burden on patients, providers, and health systems to obtain reimbursement for medically indicated treatments^15^. Moreover, concerns about data privacy, model transparency, and algorithmic bias must all be considered when integrating operational AI tools within healthcare^16^.

Similar economic pressures that have reshaped other industries are now leading health systems to the same precipice, wherein redesigning healthcare delivery may be a requirement to remaining solvent and meeting healthcare demands. Increasingly, health systems are becoming responsible for integration, validation, and monitoring of clinical AI tools. This presents an apt opportunity to use experiences from other industries to shape an operational AI roadmap for healthcare.

## Lessons from other industries

Many industries outside of healthcare have already adopted AI tools to optimize operations^17–25^. A literature search was performed to identify major industrial sectors that have integrated AI into their operational workflows, and then a focused review of each industry was performed to identify relevant applications with parallels to healthcare. A list of industry applications and parallel healthcare domains is outlined in Table 1. Here, we highlight several examples that have the potential for immediate impact in the healthcare sector.

**Table 1:** Industry-based operational AI applications with healthcare parallels.

Industry	Industry AI application	Parallel domain in healthcare	Complexity	Citation(s)
Agriculture	Moisture/Thermal Sensing	Medical Device Monitoring	Moderate	^17^
Aviation	Air Traffic Management	Hospital Flow and Throughput	Moderate	^18^
	Aircraft Maintenance Prediction	Biomedical Equipment Maintenance	Low	^39^
	Passenger/Flight Scheduling	Clinic or Operating Room Scheduling	Low	^19^
	Pilot Mental Health Monitoring	Patient or Health Provider Mental Health Monitoring	Low	^40,41^
	Weather Forecasting	Hospital Surge Forecasting	High	^37,38^
Finance	Financial Advisory Queries	Patient Medical Advisory Queries	Low	^43,44^
	Market Forecasting	Utilization Forecasting (e.g., Admission Risk, ICU Transfer)	Moderate	^20^
Human Relations	Customer Service	Patient Communication and Education	Low	^21,47,48^
Information Technology	Cybersecurity	Medical Data Security	Low	^22^
	Internet Search, Text Completion	Chart Summarization, Documentation Templating	Low	^52–55^
	Recommendations and Marketing	Preventative Health Screening, Specialist Referral	High	^11,49^
Manufacturing	Energy Consumption Forecasting	Hospital Resource Forecasting	Moderate	^23,34^
	Quality Control	Medical Error Detection	Moderate	^23,33^
Retail	Consumer Sentiment Tracking	Patient Health Monitoring	Moderate	^26^
	Supply Chain Optimization	Hospital Flow Optimization	Moderate	^28–32^
	Targeted Advertising	Targeted Medical Testing and Diagnostics	High	^24^
Transportation	Autonomous Vehicles	Hospital Transport, Supply Delivery	High	^25^

Industry-based AI applications are listed with parallel healthcare domains, implementation complexity, and corresponding literature citations. Low complexity indicates industry applications with direct parallels to healthcare or that are actively being implemented; moderate complexity indicates applications that would require additional healthcare-specific testing prior to implementation; high complexity indicates applications that would require further research or development for healthcare use.

*AI* artificial intelligence, *ICU* intensive care unit.

### Retail and manufacturing

The retail and manufacturing sectors have long employed AI systems to forecast product demand, manage inventory, optimize supply chains, and improve quality control^26–29^. Inventory management systems and AI forecasting have allowed the German online retailer Otto to reduce their held inventory by 90%^30^. Similar systems have been implemented by large U.S. retailers^31,32^, as well as 42% of small and medium retail enterprises in North America^33^. Similar models could be implemented to forecast hospital capacity, track patient flow, optimize the medical supply chain, and reduce resource waste. AI-driven route optimization systems are common among delivery companies such as the United Parcel Service (UPS), Amazon, and DHL, a global logistics provider. Estimates suggest that UPS’s On-Road Integrated Optimization and Navigation system has saved the company $300 to $400 million annually on fuel costs, and has reduced CO_2_ admissions by nearly 100,000 metric tons annually^34^. Similar systems in healthcare can help improve field response times for emergency services, help navigate hospital capacity surges, and manage large-scale emergencies. In quality improvement, Boston Scientific invested $50,000 to save $5 million by converting a rule-based quality control system for coronary stent production to an AI model, while simultaneously achieving higher performance^35^. Analogous models in healthcare can optimize automated treatment and medication checks, which currently rely on rule-based systems, with potential for similarly high return on investment if implemented at scale.

### Aviation

Similar to healthcare, aviation is a service industry that prioritizes safety, efficiency, and user experience. Aviation companies have employed AI for air traffic management and safety, maintenance scheduling, passenger support, and cybersecurity^18,19,36^. Recent breakdowns in air traffic control infrastructure have demonstrated just how reliant the industry is on technology to maintain safe air travel. Delta and other airline companies have used AI prediction tools to forecast weather disruptions, improve safety, and minimize flight delays^37^, and the National Weather Service has reported that AI systems have improved its forecast accuracy by up to 10%^38^. Similar systems can be used to optimize healthcare scheduling, reduce operating room downtime, and forecast hospital capacity. Southwest and others have used AI models to predict required aircraft maintenance^39,40^, which can be applied to biomedical equipment or expanded to recommend health maintenance exams for overdue patients. AI systems have even been proposed to help monitor mental health among pilots and air traffic controllers^41^, a practice that could be applied to patients and health providers. AI technologies have also been used to address staffing shortages among aircraft maintenance technicians^42^, which highlights a potential avenue for addressing labor shortages within healthcare.

### Customer relations

Financial companies maintain a customer-advisor relationship akin to the patient-provider partnership, and many in the financial sector have begun implementing AI-powered systems to provide financial advisory services^43–45^. Crédit Mutuel, a French banking cooperative, implemented a language-based AI tool capable of providing high-quality response to 85% of customers’ financial questions, cutting their average query resolution time from 3 min to 1 min^35^. PenFed, the second-largest U.S.-based credit union, has embraced a similar technology^46^. The fashion industry is another example, where AI systems have been used to drive customer loyalty, gauge consumer sentiment, and elicit feedback^47,48^. Beyond providing chatbots for customer service, brands have also developed AI systems for brand promotion and sentiment tracking on social media^49^. Similar technologies can be used in healthcare to track health trends, improve population health initiatives, and screen for depression and mood disorders.

The potential of AI systems to improve clinical operations has been the most evident in the field of patient communication. Several recent studies have reported on the use of AI tools to improve inbox management, patient education, and clinical documentation^50–55^. Despite these advances, healthcare still lags behind other industries in this area of AI integration.

## An AI roadmap for healthcare operations

Other industries have demonstrated success with AI tools in a variety of operational applications. However, financial barriers, rigid health system structures, and cultural inertia make a similar transformation more difficult to achieve in healthcare^56,57^. Furthermore, healthcare-specific factors including liability, ethical use, long-term monitoring, and data security are all serious concerns that must be addressed before AI can be widely adopted in healthcare^57,58^. Health systems can take several steps to begin addressing these concerns and facilitate effective AI operations in their clinical workflows.

### Overcoming the productivity paradox

Paradoxically, the introduction of new clinical technologies has historically increased overall healthcare spending, a trend that has been demonstrated for many general purpose technologies across various sectors^59^. Achieving effective performance and return on investment requires health systems to pair investments with evidence generation and close evaluation to identify early successes and failures. Increasingly subjected to external financial pressures due to market consolidation and changing federal funding, health systems must measure their return on investment from operational AI systems. Unfortunately, recent evidence suggests only 61% of U.S. hospitals performed any local performance evaluation of AI models prior to deployment, suggesting that many health systems lack the expertise or infrastructure required to validate AI performance or assess the value of AI investments^60^. National AI health collaboratives such as the Health AI Partnership, the Coalition for Health AI, and the American Medical Association’s newly launched Center for Digital Health and AI can empower resource-limited health systems to leverage peer expertise at other institutions, troubleshoot common problems, and disseminate findings related to operational AI tools^61^.

Other industries have overcome this productivity paradox by redesigning operational processes, structures, and even the culture of the workplace^62^. Rather than forcing AI tools to fit into our current clinical workflows, we must re-design our health systems to generate value through AI. For example, in nearly every health system, the frequency of follow-up appointments is left to individual physicians and there is high variability and little evidence guiding the safety of these decisions. Reallocating appointment frequency based on need (as estimated by AI) could substantially increase capacity and reduce wait times. One study found that reducing the frequency of follow-up by a single visit per year could save $1.9 billion nationally^63^. The implementation of such AI solutions would require redesigning health systems around AI rather than simply reworking AI to support existing practice patterns.

### Aligning AI operations with research

Much like how research and development is closely tied to operations in the private sector, academic medical centers must closely integrate AI operations and research^64^. Many institutions have duplicated structures for AI operations and research, even as these two areas of AI expertise become increasingly co-dependent. Given slated reductions in federal research funding, this places researchers at risk of losing access to key AI infrastructure. In the absence of vibrant research ecosystems, health systems run the risk of being reliant on vendors without local expertise to fill this gap. Both the duplication and gap in resources could be resolved through better alignment between AI operations and research within health systems. The learning health system model provides the perfect framework for doing so, ensuring that operational goals are paired with high-quality evidence generation^65^. An integrated AI workflow for both research and operations would also allow health systems to address key concerns surrounding healthcare data privacy and security within a singular, closed system, thereby reducing risk for data leaks or unauthorized access. Integrating research with operations would also expedite research to help maintain alignment with the rapid pace of AI development.

### Stronger AI literacy among health systems

Health system information technology (IT) departments, built to support operational tools such as EHRs, often lack AI expertise. As both predictive and generative AI applications become more widely available, implementation decisions reflecting AI capabilities can only occur with an informed workforce. For example, AI agents provide a mechanism to streamline hundreds of team-based workflows, creating the potential to save time and money. However, no single team at a health system has the capacity to build the AI agents needed to fill all the operational gaps. This requires distributed expertise across many teams, which is reliant on team members to have high AI literacy. Inclusion of healthcare professionals in AI literacy initiatives is critical, as point-of-care providers are increasingly asked to integrate AI tools into their clinical workflows.

### Policy and regulation

Currently, regulatory guidance on health AI technologies remains limited. FDA in the U.S., the Medicines and Healthcare products Regulatory Agency in the UK, the European Union, and the National Medical Products Administration in China have all moved toward the regulation of clinical AI software as a medical device^66,67^. However, these guidelines remain limited in scope and are largely focused on a “high-risk” subset of AI technologies^68^. As a result, health systems that serve as the site of AI implementation have been forced to play a major role in the evaluation and oversight of clinical AI systems with only limited regulatory guidance. Clearer federal regulation with more granular details on best practices for assessment and monitoring, including requirements for AI developers to disclose training data, would go a long way in supporting health systems in effective AI integration.

## Conclusion

Many other industries have found success in incorporating AI into operational workflows, and there is vast potential in adopting parallel technologies in healthcare. Effective implementation of AI in healthcare will require redesigning our health systems around AI, merging of AI operations and research, robust evaluation, greater AI literacy, and effective policy and regulation.

## Data Availability

No datasets were generated or analyzed during the current study.
